# An Occult Malignancy Behind a Demyelinating Disease: POEMS Syndrome

**DOI:** 10.1177/2324709616673389

**Published:** 2016-10-18

**Authors:** Saberio Lo Presti, Prashanth Kanagarajah, Daniela Pirela, Diana Morlote, Mike Cusnir

**Affiliations:** 1 Mount Sinai Medical Center, Miami Beach, FL, USA

**Keywords:** POEMS, demyelination, plasmacytoma, paraneoplastic syndrome

## Abstract

We report a case of a 38-year-old man presenting with bilateral lower extremity weakness and paresthesias that progressed during a 4-month period to severe polyneuropathy forcing the patient to be bed bound. Throughout his multiple hospitalizations, he was treated erroneously for chronic inflammatory demyelinating polyneuropathy, without significant improvement in his symptoms. In addition, he developed hepatosplenomegaly (organomegaly); endocrinopathies such as diabetes mellitus, central hypogonadism, and hypothyroidism; monoclonal spike evidenced in the serum electrophoresis; and hyperpigmentation of skin, altogether consistent with POEMS syndrome. During his last hospitalization he developed excruciating pain on his left hip, and imaging revealed the presence of a 9 × 6 cm osteolytic mass with sclerotic rim in the left acetabulum. Biopsy of the mass confirmed an isolated IgG lambda plasmacytoma. The patient received radiation to his left acetabular lesion followed by left hip replacement. Subsequently, the patient underwent autologous bone marrow transplant. Eighteen months after his initial presentation, he had satisfactory clinical response and is functional without significant limitations. POEMS syndrome is a rare paraneoplastic syndrome secondary to an underlying plasma cell disorder, which can oftentimes be overlooked and misdiagnosed. The median age of presentation is 51 years, and only 31% of the cases occur in fairly young patients under the age of 45 as evidenced in this case. As clinicians, we should be aware of the constellation of features associated with POEMS syndrome and be able to recognize them promptly.

## Introduction

POEMS syndrome is a rare paraneoplastic syndrome secondary to an underlying plasma cell disorder.^1^ This acronym was first coined in 1980 by Bardwick referring to the most common features seen in this particular syndrome: Polyneuropathies, Organomegaly, Endocrinopathies, Monoclonal spike, and Skin changes.^1^ However, throughout the last decades other clinical features were also recognized to be part of this syndrome. These additional features include papilledema, extravascular fluid overload, sclerotic bone lesions, thrombocytosis/erythrocytosis, elevated vascular endothelial growth factor (VEGF), and abnormal pulmonary function tests.^2^ The incidence of POEMS syndrome is unknown in the United States, but in Japan it is estimated to be around 0.3 per 100 000 habitants.^3^

In the Mayo Clinic series of 99 cases, the median age of presentation was 51 years (range = 30-83), and in up to 31% of the cases this condition was seen in individuals younger than 45 years. In addition, it was predominantly seen in males representing 63% of the cases.^4^

Being rare, it is not a commonly sought after diagnosis. Clinical presentation can vary and identification can be cumbersome. We describe a young patient diagnosed with POEMS syndrome after presenting with progressive deterioration of his neurological deficit despite conventional therapy for what was assumed to be chronic inflammatory demyelinating disease (CIDP).

## Case Report

We present a previously healthy 38-year-old Hispanic male admitted with progressive lower extremities paresis and paresthesia. Four months prior to admission, he developed paresthesia in his toes, which progressed to symmetrical ascending lower extremity weakness and unsteady gait. Time from symptom onset to presentation was 3 weeks. Review of systems was positive for impotence, polyuria, polydipsia, and skin changes, namely hyperpigmentation and hypertrichosis. He was initially treated with a 5-day course of intravenous immunoglobulin (IVIG) for suspected Guillain-Barré syndrome based on his clinical presentation and the presence of albumin cytologic dissociation found on the cerebrospinal fluid analysis. Despite the IVIG, his symptoms did not improve significantly. He was later transferred to recuperate in rehabilitation, and once his symptoms stabilized, he was discharged home with outpatient follow-up. He relapsed 4 weeks after discharge, this time reporting paresthesia of his hands as well. Nerve conduction velocities revealed a demyelinating pattern, and a repeated lumbar puncture showed oligoclonal bands. Additionally, antibodies anti-Hu and myelin basic proteins were requested as part of the workup for paraneoplastic encephalomyelitis and multiple sclerosis with negative results. In light of the relapsing nature of his presentation, he was treated for suspected CIDP with plasmapheresis, IVIG, and a short course of intravenous steroids, again with only partial improvement.

Four months after the initial presentation, his neurologist found a positive monoclonal spike on serum protein electrophoresis and referred him for hospitalization. This time, we encountered a patient with profound lower extremity weakness predominantly proximal, more evident on the left lower limb. His neurological examination was also significant for dysesthesias, paresthesias, and hyporeflexia. Sphincter tone was preserved and there was no evidence of fasciculation, primitive neurological reflexes, or cranial nerve deficits.

**Figure 1.**
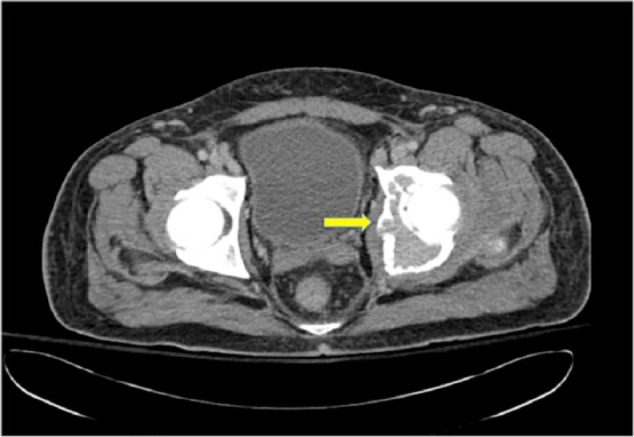
Transverse view of a pelvic CT scans showing a 9 × 6 cm osteolytic mass with sclerotic rim in the left acetabulum.

**Figure 2.**
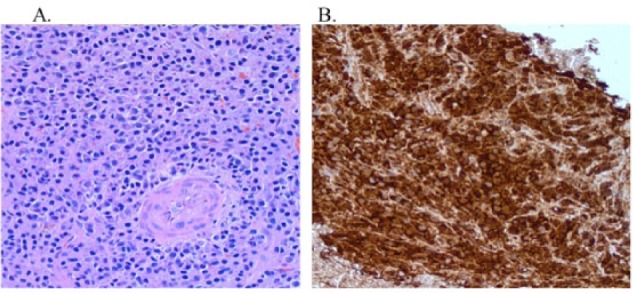
Bone biopsy of sclerotic left acetabular mass. (A) Diffuse plasma cell (hematoxylin-eosin, magnification 20×). (B) Positive stain for IgG lambda neoplastic plasma cells (magnification 10×).

This time, he exhibited poor response to repeated cycles of IVIG. He also complained of persistent abdominal and left inguinal pain with exquisite point tenderness over the left hip. Computed tomography (CT) abdomen/pelvis revealed the presence of a 9 × 6 cm osteolytic mass with sclerotic rim in the left acetabulum (Figure 1), hepatosplenomegaly in the settings of a fairly normal hepatic profile, small pleural effusions and mild ascites. CT-guided biopsy of the mass revealed the presence of plasma cells, consistent with a single plasmacytoma which stained positive for IgG lambda (Figure 2). Repeated serum protein electrophoresis with immunofixation confirmed a monoclonal spike IgG lambda (0.64 µg/dL). Further workup revealed the presence of new-onset hypothyroidism, diabetes mellitus, and central hypogonadism with a normal sellar MRI (magnetic resonance imaging). In addition, VEGF level was found increased to 5575 pg/mL (normal range = 31-86 pg/mL). All these clinical and laboratory data were suggestive of POEMS syndrome.

After establishing the diagnosis, the patient received radiation to his left acetabular lesion followed by left hip replacement. Subsequently, because of his young age and devastating neuropathies, he underwent treatment with autologous bone marrow transplant. The patient improved clinically in terms of his neuropathies, which stabilized around 2 months after bone marrow transplant, and he had satisfactory radiologic response with a FDG-PET (fluorodeoxyglucose positron emission tomography) scan showing no new lesions.

His postoperative recovery was complicated with cholecystitis requiring cholecystectomy and a subsegmental pulmonary embolism treated with oral anticoagulant. Eighteen months after his initial presentation, he continues to maintain a good performance status (Eastern Cooperative Oncology Group [ECOG] 0). The patient is able to ambulate freely, although requires bilateral orthotic devices on his lower extremities to treat foot drop. His neuropathic pain is controlled on pregabaline. In terms of his endocrinopathies, he is being treated with thyroid hormonal replacement and remains biochemically euthyroid. His sexual dysfunction is managed with testosterone supplementation and tadalafil. His most distressing problem seems to be chronic diarrhea in the setting of unrevealing upper and lower endoscopies and is treated symptomatically along with dietary changes.

In terms of laboratory findings, the immunoglobulin levels remained essentially normal and there was no evidence of erythrocytosis or thrombocytosis. On his physical examination, he looked euvolemic and no organomegaly was appreciated. His neurological examination revealed bilateral foot drops and he still exhibited dysesthesias on his feet.

## Discussion

This case illustrates the challenge of identifying POEMS syndrome and how the constellation of symptoms may have a delayed presentation. POEMS syndrome is frequently misdiagnosed with other demyelinating diseases such as CIDP, monoclonal gammapathy of unknown significance, and immunoglobulin light chains amyloid neuropathy.

Polyneuropathies is the debuting symptom in up to 50% of patients of the cases and more than half of them are initially treated incorrectly as CIDP. Both conditions present with symmetrical polyneuropathies, physiological evidence of demyelization, and albumin cytological dissociation.^3^ However, these differential diagnoses could be excluded early with the aid of a complete radiographic assessment of the bones, measurement of VEGF, careful analysis of bone marrow biopsy, and nerve conductive studies.^1,3^

The exact causative mechanism of demyelination in POEMS syndrome is unclear. The protein VEGF seems to play a central role in the pathophysiology of this condition, and it is an important surrogate marker of disease.^5^ However, the mixed results in clinical trials with bevacizumab, a monoclonal antibody that targets this protein, exposes a more complex underlying mechanism of the disease,^6^ which has increased the interest toward IL-1B and IL-6, both stimulators of VEGF.^7^

In order to make the diagnosis of this syndrome, recent updated clinical and laboratory data were established. It is required to meet 2 major criteria or 1 major and 1 minor criterion (Table 1).^1^

**Table 1.** Diagnostic Criteria of POEMS Syndrome.

Mandatory major criteria	1. Polyneuropathy (typically demyelinating)
	2. Monoclonal plasma cell proliferative disorder (almost always λ)
Other major criteria (one required)	3. Castleman disease
	4. Sclerotic bone lesions
	5. VEGF elevation
Minor criteria	6. Organomegaly (splenomegaly, hepatomegaly, or lymphadenopathy)
	7. Extravascular volume overload (edema, pleural effusion, or ascites)
	8. Endocrinopathy (adrenal, thyroid, pituitary, gonadal, parathyroid, and pancreatic)
	9. Skin changes (hyperpigmentation, hypertrichosis, glomeruloid hemangiomata, plethora, acrocyanosis, flushing, and white nails)
	10. Papilledema
	11. Thrombocytosis/polycythemia
Other symptoms and signs	Clubbing, weight loss, hyperhidrosis, pulmonary hypertension/restrictive lung disease, thrombotic diatheses, diarrhea, low vitamin B_12_ values

It is also important to distinguish POEMS from other plasma cell proliferative disorders, not only to implement a more effective treatment but also to avoid the toxicity from more deleterious therapies. It is important to consider that one third of POEMS syndrome patients do not have plasma cells on their iliac crest biopsy. These less common cases are found to have a solitary plasmacytoma as was seen in our case or multiple plasmacytomas.^8^

The median survival of these patients is close to 14 years with appropriate medical therapy. There are clinical features associated with worse prognosis; clubbing of the finger nails, extravascular volume overload, respiratory symptoms, and pulmonary hypertension.^9^

Finally, the management of this condition depends on the extent of the disease. Radiation therapy has shown positive results in clinical trials when there is absence of bone marrow involvement and less than 3 bone lesions. Conversely, once the disease is advanced, chemotherapy is necessary. The most studied therapy consists of alkylator therapy (low or high dose) with stem cell transplant. In addition, chemotherapy with melphalan and dexamethasone has proven to result in positive neurological, hematological, and VEGF response.^10^

## Conclusion

Early diagnosis of POEMS syndrome is important to implement the most appropriate management. Clinicians should have a low threshold of suspicion in patients presenting with refractory demyelinating diseases, since almost half of the cases debut with polyneuropathies. The constellation of symptoms characteristics of POEMS syndrome can often have a delayed presentation deferring its recognition, which can decrease the benefits of implementing therapies early on. A good clinical assessment combined with laboratory data and radiologic assessments of bones are crucial to establish the accurate diagnosis.
